# Apple polyphenol extract improves insulin sensitivity *in vitro* and *in vivo in animal models of insulin resistance*

**DOI:** 10.1186/s12986-016-0088-8

**Published:** 2016-04-30

**Authors:** Manuel Manzano, María D Giron, José D. Vilchez, Natalia Sevillano, Nuri El-Azem, Ricardo Rueda, Rafael Salto, Jose M. Lopez-Pedrosa

**Affiliations:** Strategic R&D, Abbott Nutrition Research & Development, Camino de Purchil, 68, Granada, Spain; Biochemistry and Molecular Biology II, University of Granada, Granada, Spain

**Keywords:** Apple polyphenol extract, Insulin sensitivity, Blood glucose uptake, Skeletal muscle, Diabetes

## Abstract

**Background:**

Apple polyphenols could represent a novel nutritional approach in the management and control of blood glucose, especially in type 2 diabetics. The aim of this study was to test the therapeutic potential of an apple polyphenol extract (APE) in an insulin-resistant rat model and to determine the molecular basis of insulin sensitivity action in skeletal muscle cells.

**Methods:**

Acute effect of APE on the postprandial hyperglycemic response was assayed in 15 week old obese Zucker rats (OZR), by using a meal tolerance test (MTT). The ability of APE to improve whole peripheral insulin sensitivity was also assayed in a chronic study by using the euglycemic-hyperinsulinemic clamp technique. To elucidate the molecular mechanisms, rat L6 myotubes were used. Glucose uptake was measured by using 2-[3H]-Deoxy-Glucose (2-DG) and specific inhibitors, as well as phosphorylation status of key kinases, were used to determine the implicated signaling pathway.

**Results:**

*In vivo* study showed that nutritional intervention with APE induced an increase of insulin sensitivity with an increase of glucose infusion rate (GIR) of 45 %. Additionally, i*n vitro* results showed a synergistic effect between APE and insulin as well as increased glucose uptake through GLUT4 translocation in muscle cells. This translocation was mediated by phosphatydil inositol 3-kinase (PI3K) and peroxisome proliferator-activated receptor-gamma (PPARγ) signaling pathways.

**Conclusions:**

As a whole, this study describes the mechanisms involved in the insulin sensitizing effect of APE, which could be considered a promising ingredient for inclusion in nutritional products focused on the management of chronic diseases such as diabetes.

## Background

Diabetes, in particular type 2 diabetes, is one of the major worldwide health problems. To avoid the increasing prevalence of diabetes, it is imperative to focus on the prevention of insulin resistance, which precedes the onset of diabetes. During this stage, different approaches have focused on lowering intestinal carbohydrate digestion and absorption through the inhibition of α-amylase and α-glucosidase, increasing insulin secretion and glucose tissue disposal (insulin sensitizing agents) and decreasing hepatic glucose production through the inhibition of gluconeogenesis and glycogenolysis in the liver [1, 2]. In this framework, nutritional dietary components have performed well in clinical practice and are showing optimal results in the prevention of diabetes progression.

Among dietary components that could exert a positive and beneficial effect in the control of blood glucose, fruits and vegetables have shown promising results. Indeed, epidemiological evidence suggests that a diet rich in fruits and vegetables may decrease the risk of chronic diseases such as cardiovascular disease and diabetes [3]. Moreover, interventional clinical trials support the role of polyphenols, flavonoids and carotenoid [4] rich foods in the prevention of chronic diseases associated with oxidative stress such as type 2 diabetes.

Apples are one of the most consumed sources of polyphenols in the Western diet containing mainly quercetin, catechin, proanthocyanidin, gallic acid, phloridzin and chlorogenic acid [5]. Apple polyphenols have shown strong antioxidant activity [6], anti-carcenogenic effects [7] and a high capacity to reduce lipid oxidation [8], as well as hipocholesterolemic effects [9]. Regarding the potential antidiabetic effects of apple polyphenols, high intake of quercetin, chlorogenic acid and phloridzin has been associated with reduced postprandial hyperglycemia primarily by inhibiting intestinal glucose absorption [10]. Glucose is absorbed by the intestine mainly through sodium-glucose linked transporter 1 (SGLT1) and glucose transporter 2 (GLUT2) [11]. Many phenolic compounds, including apple polyphenols, have shown the ability to inhibit these transporters. In addition to inhibition of glucose transport, these polyphenols also have the ability to directly inhibit enzymes responsible for the hydrolysis of carbohydrates at intestinal level (α-amylase and α-glucosidase) [10], further reducing intestinal glucose absorption.

However, the potential effects of muscle insulin sensitivity associated with apple polyphenols remains unknown. Therefore, the present study was designed to evaluate the effect of an apple polyphenol extract (APE) on insulin sensitivity in obese Zucker fatty fa/fa rats (OZR, an insulin resistant model) as a novel antihyperglycemic mechanism to counteract diabetes progression. Moreover, the molecular basis of the insulin sensitivity action of APE in cultured skeletal muscle cells as an insulin-responsive model was also evaluated. Our results show that the antihyperglycemic effects of APE are mediated not only by inhibiting intestinal glucose absorption but also by stimulating muscle insulin sensitivity through a mechanism that involves increased expression of glucose transport proteins.

## Methods

### Evaluation of the antidiabetic effect of APE in OZR

#### Animals

Fourteen week old, obese male OZR were obtained from Charles Rivers Laboratories (Orleans Cedex, France). They were individually housed under a 12-h cycle of light and dark at 21 °C. All experimental procedures were carried out according to the ethical guidelines for animal experimentation provided by the Spanish National Research Council (RD 1201/2005 October 10).

### Acute effect of APE on the postprandial glycemic response during the time course of a Meal Tolerance Test (MTT)

Twenty rats were assigned into two groups: Control (*n* = 10) and APE groups (*n* = 10). The control group was orally treated with a solution containing maltodextrin (MD, DE 21.5, Cerestar, Spain) at the dose of 1 g/kg body weight (bw). The APE group was given an oral dose of MD containing APE at the dose of 150 mg APE/kg bw. APE was purchased from Exxentia (Spain) and the polyphenolic concentration of this extract was 57.5 %, underscoring the amount of phloridzin (9.9 %), chlorogenic acid (15.8 %) and quercetin (0.4 %). Blood samples were obtained via tail vein at baseline and at 30, 60, 90 and 120 min postprandial for glucose and insulin analysis.

In order to investigate the second-meal effect after the ingestion of APE at breakfast, a second meal tolerance test was performed by administering only MD at the dose of 1 g/kg bw after 270 min from the first gavage. Blood samples were also collected at baseline and 30, 60, 90, 120, 180 and 240 min for glucose and insulin analysis.

### Ex-vivo α-glucosidase activity of APE

α-glucosidase inhibitory properties of APE were assayed in intestinal mucosa isolated from OZR following Dahlqvist methodology [12]. Maltase, isomaltase and sucrase activities were determined using maltose, isomaltose and sucrose as substrates. Results were expressed as the concentration of APE required to achieve 50 % of inhibition (IC50).

### Chronic effect of APE extract on insulin sensitivity

The effect of APE on whole body insulin sensitivity was performed on 15 week old OZR. Twenty OZR were weighed and divided into two experimental groups (Control and APE) of ten animals. The control group received the standard purified rodent diet (AIN-93 M) [13] *ad libitum* for five weeks. The APE group received the standard diet supplemented with APE (3 g/kg diet) during the same period. Individual body weights were recorded at the beginning of the study and at weekly intervals thereafter. The food consumption of each rat was determined twice per week.

Four weeks prior to the end of the feeding study, an additional MTT assay was performed on both study groups using MD in fasting conditions.

The insulin sensitizing effect of APE was determined one week later using a modified euglycemic-hyperinsulinemic clamp technique developed by De Fronzo et al. [14]. In brief, overnight-fasted rats were anesthetized with sodium pentobarbital (50 mg/kg bw) (Pentothal, Abbott Laboratories, Madrid, Spain). Two catheters were placed in the right jugular vein for insulin and glucose infusion. Another catheter was placed in the left carotid artery for blood sampling. A tracheotomy was performed to allow for tracheal clearing. After approximately 40 min of surgery, arterial blood samples were collected for determination of glucose and insulin basal concentrations. At time 0, human insulin (Humulin R; Eli Lilly,Indianapolis, IN) was infused at a concentration of 15 mU/kg per minute. Blood samples were subsequently drawn at five-minute intervals for determination of blood glucose (Precision G Medisense, Bedford, Massachusetts, USA). An infusion of 30 % glucose was adjusted to maintain blood glucose at 100 mg/dL. Steady state was ascertained when a fixed glucose infusion rate (GIR) maintained blood glucose measurements constant for at least 30 min.

### Analytical procedures

Blood glucose was determined using Precision PCx glucose meter equipment (Abbott Laboratories). Serum insulin concentration was measured by enzyme immunoassay (Mercodia AB, Uppsala, Sweden) using rat insulin as standard. Serum biochemical markers were determined using an Alcyon C-3500 autoanalyzer (Abbott Laboratories).

### Evaluation of the insulin sensitivity mechanisms of APE in L6 myotubes

#### Cell culture

L6.C11 rat skeletal muscle myoblast line (ECACC n° 92102119) and CHO-k1 (ATCC no. CCL-61) cells were grown in Dulbecco’s modified Eagle’s medium (DMEM) containing 10 % (v/v) fetal bovine serum (FBS), 2 mM glutamine plus 100 units/mL penicillin and 0.1 mg/mL streptomycin (Sigma, St. Louis, MO, USA) in an atmosphere of air/CO_2_ (95:5) and maintained at subconfluent densities in growth media. L6 myoblasts were differentiated into myotubes by exchanging the growth medium with a differentiation medium consisting of DMEM containing 2 % (v/v) fetal bovine serum for 5–6 days (>50 % fusion into multinucleated myotubes).

#### 2-Deoxy-[^3^H]D-glucose (2-DG) uptake

Cells were grown in 48-well plates (Corning, NY, USA). They were differentiated into myotubes and then incubated in serum-free medium for 18–21 h. Treatments were performed in serum-free medium unless otherwise indicated. Triplicate measurements of 2-DG (Perkin Elmer, Waltham, MA, USA) uptake were taken after 10 min of incubation following the method described by Yonemitsu et al. [15].

#### Subcellular fractionation

Membrane fractions from myotubes were prepared as described [15, 16]. 5′-nucleotidase and cytochrome c reductase activities were assayed as marker enzymes for plasma membranes and low-density microsomes, respectively [17].

#### Glucose transporter 4 (GLUT4) protein analyses

L6 myotubes membrane fraction proteins were electrophoresed in 10 % (w/v) SDS-PAGE and processed for western blot with anti-GLUT4 antibodies (Biogenesis, Poole, UK). Immunoreactive bands were visualized by chemiluminescence and quantified with NIH Image Software. Results were normalized with the band intensity of actin (Developmental Studies Hybridoma Bank, Iowa City, IA, USA) or caveolin-1 (Cell Signaling Technology, Beverly, MA, USA).

#### Signaling pathways analyses

L6 myotubes were preincubated with different inhibitors implicated in distinct signaling pathways leading to GLUT4 translocation: wortmannin (100 nmol/L) (an inhibitor of the PI3K), PD 98059 (30 μmol/L) (an inhibitor of the ERK1/2 phosphorylation) and GW9662 (10 μmol/L) (an inhibitor of PPARγ). The inhibitors were added 30 min before the incubation with APE (25 μg/mL) and maintained during the treatments (2 h).

Protein phosphorylation analysis were carried out as described in [16]. Briefly, L6 myotubes were incubated in FBS-free medium for 18–24 h and then treated with APE (25 μg/mL) or rosiglitazone (10 μmol/L) in serum-free medium. After treatment, plates were flash frozen in liquid nitrogen and scraped with 50 μL/60 mm/plate of cold 30 mmol/L Tris-HCl pH 7.4, 25 mmol/L NaCl, 1 % (v/v) Triton X-100, 0.1 % SDS, 10 mmol/L sodium fluoride, 10 mmol/L sodium pyrophosphate, 1 mmol/L sodium orthovanadate, 1 mmol/L EGTA, 20 nmol/L okadaic acid, 10 μg/mL aprotinin, 10 μg/mL leupeptin, 10 μg/mL pepstatin. After 10 min on ice, extracts were centrifuged at 13,000 *×g* for 10 min at 4 °C. For the study of PPARγ expression, nuclear extracts were obtained in accordance with Giron et al. 2008.

For the western blots analysis, proteins (25 μg) were separated by SDS-PAGE and immunoblotted with antibodies against PPAR-γ (Santa Cruz Biotechnology, Santa Cruz, CA, USA) and histone H3 (Epitomics, Burlingame, CA, USA). The immunoblots were developed by chemiluminescence detection.

The expression plasmids pSV SPORT PPARγ1 and pSV SPORT PPARγ2 and the PPAR responsive element-driven luciferase reporter vector PPRE X3-TK-Luc PPRE X3-TK-Luc were kindly provided by Dr. Bruce M. Spiegelman (Dana-Farber Cancer Institute, Harvard Medical School, Addgene plasmids 8886, 8862 and 1015). CHO-k1 cells were transiently transfected with either pSV SPORT PPARγ1 or pSV SPORT PPARγ2, the PPRE X3-TK-Luc and the control plasmid pRL-TK. After 24 h, cells were treated with rosiglitazone (10 μmol/L) or APE (25 μg/mL) and cultured for 4 h in DMEM containing 2 % (v/v) Charcoal/dextran stripped FBS (Sigma). The cells were then used for dual luciferase reporter gene assay.

### Statistical analyses

Data are presented as mean ± standard error of the mean (SEM). In the case of the *in vivo* study, two-way repeated measurements ANOVA was used to analyze the data for body weight, intake, glycemic and insulinemic responses. *A posteriori* comparisons were done using the Bonferroni test. The remaining data was analyzed using the unpaired Student *t* test. All statistical analyses were performed using GraphPad 4.0 software.

Statistical analysis for *in vitro* studies was performed by one way ANOVA followed by the Tukey test as appropriate. *P* < 0.05 was considered statistically significant.

## Results

### Evaluation of the antidiabetic effect of APE in OZR

#### Acute effect of APE on the postprandial response

**Fig. 1:**
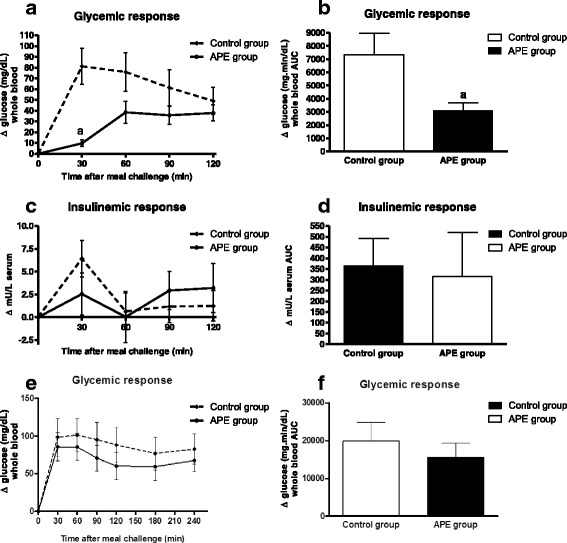
Acute effects of APE (dose of 150 mg/kg bw) on the postprandial glycemic (**a**) and insulinemic (**c**) responses of maltodextrin in OZR and on the respective areas under the curve (**b**, **d**). Acute effects of APE (dose of 150 mg/kg bw) on the second-meal effect. Blood glucose (**e**) and area under the curve (**f**) after a first load of 150 mg APE/kg b.w. at 8:00 in the morning (simulating breakfast meal) and after a second load 1 g maltodextrin/kg bw (simulating lunch meal) 5 h later. Results are expressed as mean ± SEM, *n* = 10 in each group. (*a*) *p* < 0.05 compared with control group

Figure 1 shows plasma glucose and serum insulin levels following an oral meal challenge using MD in OZR. At 30 min post-gavage (Fig. 1a), the glucose concentration was significantly lower in the group acutely administered APE (150 mg/kg body weight) as compared to the group treated only with MD (1 g/kg body weight). Incremental area under curve (AUC) of glycemic response (Fig. 1b) was also significantly lower after receiving APE as compared with the group that only received MD. Nonetheless, there were no differences in the insulinemic response between both groups (Fig. 1c and d).

Moreover, we investigated whether APE ingestion at breakfast could improve glycemic and insulinemic responses after lunch, referred to as the second-meal effect. The glycemic response after the second oral challenge using only MD was higher than the responses obtained after the first gavage in the two study groups (Fig. 1e). Interestingly, the group that received APE in the first MTT showed a decrease of 22 % in AUC for glucose as compared with the control group (Fig. 1f).

#### Ex-vivo α-glucosidase activity of APE

Dose-response curves of inhibitory activity of APE on intestinal α-glucosidase activities were determined and the IC50 values were calculated. APE exhibited α-glucosidase inhibitor action, with IC50 values for sucrase, maltase and isomaltase enzymes of 18.05 ± 0.15 μg/mL, 14.18 ± 0.51 μg/mL, and 12.54 ± 0.27 μg/mL, respectively.

#### Effects of APE on the regulation of insulin sensitivity

A chronic study was conducted to determine if APE hypoglycemic activity might be exerted by a different mechanism involving regulation of insulin sensitivity, Rats consuming a diet supplemented with APE (3 g extract/kg diet) for five weeks showed similar body weight gain as compared with rats consuming the control diet. There were no additional differences in daily food intake (Table 1).

**Table 1:** Effect of APE supplementation on body weight, food intake and metabolic parameters in fasting obese Zucker rats

	Groups	
	Control	APE
Initial body weight (g)	435.0 ± 8.2	436.6 ± 8.9
Final body weight (g)	473.9 ± 7.6	476.9 ± 8.9
Daily food intake (kcal)	66.03 ± 1.86	67.02 ± 1.19
Glucose (mg/dL whole blood)	181.2 ± 27.1	159.6 ± 8.5
Insulin (mU/L)	17.36 ± 1.64	11.23 ± 0.87^a^
Triglycerides (mg/dL)	482.1 ± 99.4	283.5 ± 24.7^a^
Total cholesterol (mg/dL)	219.1 ± 8.3	207.4 ± 4.3
LDL/HDL (ratio)	1.14 ± 0.09	1.07 ± 0.09

Values are means ± SEM, *n* = 10 in each group. (^a^) *vs.* Control group at *p* < 0.05

**Fig. 2:**
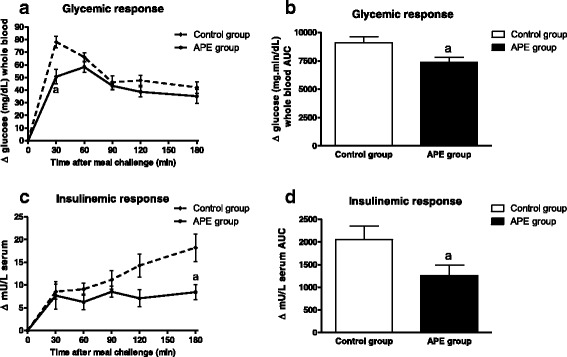
Chronic effects of APE on the postprandial glycemic (**a**) and insulinemic (**c**) responses of maltodextrin and respective areas under the curve (**b**, **d**) in OZR fed with a standard rodent diet (AIN93M) supplemented with 3 g APE/kg diet for four weeks. Results are expressed as mean ± SEM, *n* = 10 in each group. (*a*) *p* < 0.05 compared with control group

The postprandial glycemic and insulinemic responses to a MD challenge (1 g/kg bw) after a chronic consumption of APE (128 mg/kg bw) for four weeks are presented in Fig. 2. No significant differences were found between both groups regarding fasting glucose in plasma. As shown in Fig. 2a, absolute glucose levels at 30 min were lower in the APE group compared to the AIN93M fed group. Incremental area under the curve was significantly higher in the control group compared to the APE group (Fig. 2b). After four weeks of treatment, fasting insulin level was significantly lower in the APE group with respect to the control group. In addition, insulinemic response in the APE group was significantly lower in the groups fed with the APE supplemented diet as compared to the untreated group (Fig. 2c and d) during the time course of the MTT.

**Fig. 3:**
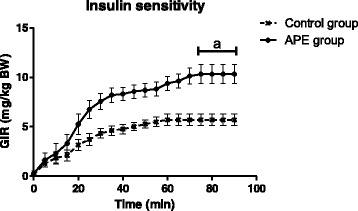
Chronic effects of APE on whole-body insulin sensitivity in OZR fed with a standard rodent diet (AIN93M) supplemented with 3 g APE/kg diet for five weeks. Results are expressed as mean ± SEM, *n* = 10 in each group. (*a*) *p* < 0.05 compared with control cells

Whole body tissue sensitivity to exogenous glucose was determined in the steady-state period of the euglycemic clamp, as expressed as glucose infusion rate (GIR) (Fig. 3). The quantity of glucose required to establish euglycemia during this period was affected by the diet. The GIR required to maintain euglycemia was significantly greater in rats fed with APE as compared to the control diet. This result signals that APE was able to stimulate glucose tissue disposal as an insulin sensitizing agent.

### Evaluation of the insulin sensitivity mechanisms of apple polyphenols extract in L6 myotubes

#### Effect of APE on 2-DG uptake

**Fig. 4:**
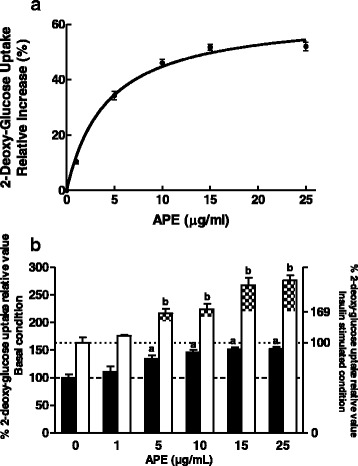
Effects of APE on 2-deoxy-glucose uptake. **a** L6 myotubes were deprived of FBS for 18 h and then incubated with increasing amounts of APE (0–25 μg/mL) for 18 h. **b** L6 myotubes were incubated with APE (1–25 μg/mL) in the absence or presence of 50 nmol/L insulin for 18 h. Results are mean ± SEM of five independent experiments. (*a*) *p* < 0.05 compared with untreated cells. (*b*) *p* < 0.05 compared with cells treated with insulin. Black bars and white bars indicate absence or presence of insulin. Patterned bar indicates synergistic effect of insulin and APE

The 2-DG assay was used to examine the effect of APE on glucose uptake in L6-myotubes. A higher glucose uptake was seen when myotubes were incubated for 18 h with increasing amounts of APE (0–25 μg/mL). 2-DG uptake was stimulated by APE, with a semi-maximal activating concentration (EC50) of 4.2 ± 0.7 μg/mL and a maximum relative increase in 2-DG uptake (Bmax) of 63.6 ± 2.3 % (Fig. 4a).

Next, to study if APE exerts any additive and/or synergistic effect on insulin stimulated glucose uptake, L6 myotubes were incubated with APE (0–25 μg/mL), insulin (50 nM) or both effectors for 18 h (Fig. 4b). The results showed that 2-DG uptake was significantly stimulated by APE in a dose dependent way. Additionally, incubation of myotubes with both effectors (APE + insulin) produced a synergistic effect on insulin-stimulated 2-DG uptake as compared to either APE or insulin alone. This synergistic effect appeared at a concentration of 5 μg/mL of APE and rose with increasing concentrations of APE, showing the highest effect at 25 μg/mL of APE.

#### Effect of APE on GLUT4 expression and translocation

**Fig. 5:**
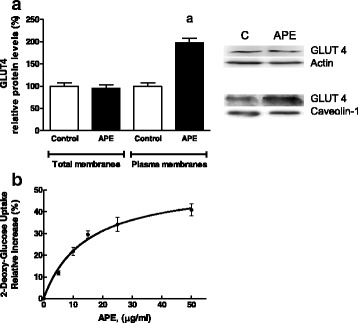
Effects of APE on GLUT4 protein levels in L6 myotubes. **a** Cells were deprived of FBS for 18 h and incubated in the absence or presence of 25 μg/mL APE for 18 h. GLUT4 protein amount was assayed by Western blot in cell lysates and plasma membrane fractions. Signal densities from control cells were assigned a value of 100 % for the transporter. Effects of APE on 2-DG uptake. **b** L6 myotubes were deprived of FBS for 18 h and then incubated with increasing amounts of APE (0–50 μg/mL) for 2 h. Results are mean ± SEM of five independent experiments. (*a*) *p* < 0.05 compared with control non-treated cells

Additionally, correlation between 2-DG uptake with the expression of GLUT4 was studied. After incubating myotubes with 25 μg APE/mL for 18 h, the amount of GLUT4 was quantified in total membranes by Western blot using actin as a control. APE-treated myotubes showed a similar content of GLUT4 to control cells (Fig. 5a).

Following this, translocation of the transporter was studied to determine if it might be a mechanism by which APE increases muscle glucose uptake. Thus, L6 myotubes were incubated in absence or presence of 25 μg APE/mL for 18 h and GLUT4 protein concentration was determined in plasma membranes using caveolin-1 as a loading control (Fig. 5a). Incubation with APE significantly increased the amount of GLUT4 in the plasma membrane fraction. Analysis of GLUT4 distribution in the different cellular membranes is the most common technique used to determine GLUT4 translocation, the rate-limiting step for glucose uptake in muscle [18]. Moreover, activation of GLUT4 translocation by 25 μg APE/mL was associated with a relative increase of approximately 30 % in 2-DG uptake (Fig. 5b).

#### Signaling pathways modulated by APE in L6 myotubes

To gain insight into the mechanism of action involved in the regulation of glucose uptake mediated by APE in muscle cells, we studied how key components of the PI3K-insulin, MAPK (ERK1/2) and PPARγ transduction pathways are modulated by this compound.

**Fig. 6:**
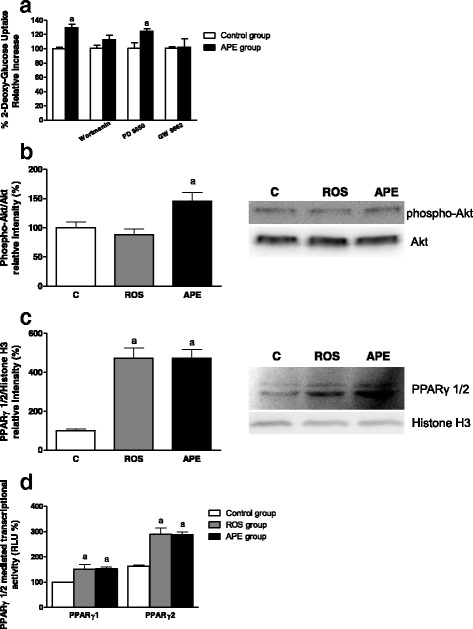
Analysis of APE effects on the signaling pathways involved in GLUT4 translocation in L6 myotubes. **a** Cells were pre-incubated with inhibitors of PI3K (100 nmol/L wortmanin), ERK1/2 (30 μmol/L PD98059) and PPARγ (10 μmol/L GW9662) and then incubated in the absence or presence of 25 μg/mL APE for 2 h. Next, 2-DG uptake was determined. **b**, **c** L6 myotubes were deprived of FBS for 18 h and then incubated with 10 μmol/L rosiglitazone (ROS) or 25 μg/mL APE for 30 min. Akt phosphorylation **b** in cell lysates, and PPARγ levels **c** in nuclear extracts were measured. **d** CHO-k1 cells were deprived of FBS for 18 h and then incubated with 10 μmol/L ROS or 25 μg/mL APE for 4 h. PPARγ 1/2 mediated transcription was measured. Results are expressed as mean ± SEM of five independent experiments. (*a*) *p* < 0.05 compared with control non-treated cells

The APE effects on glucose uptake were determined using specific inhibitors of PI3K (wortmannin), ERK1/2 (PD98059) and PPARγ (GW9662). Both wortmannin and GW9662 inhibited the increase of APE-mediated glucose uptake compared to control cells while the blockade of the ERK1/2 signaling pathway did not change APE effects (Fig. 6a).

To further confirm these results, the phosphorylation of Akt after incubation with APE (25 μg/mL) or rosiglitazone (10 μmol/L) for 30 min was studied. As expected, APE was able to significantly promote Akt phosphorylation, similar to results obtained with the specific inhibitor wortmannin (Fig. 6b).

Since the PPARγ inhibitor GW9662 was able to block the APE effects on glucose uptake, the PPARγ amount in nuclear extracts was assayed after incubation with APE (25 μg/mL) or rosiglitazone (10 μmol/L) for 30 min (Fig. 6c). Predictably, rosiglitazone, a well known PPARγ activator, induced an increase in the amount of protein in the nucleus. A similar increase was obtained after incubation with APE. To confirm the effects of APE on PPARγ signaling, a reporter assay in CHO-k1 cells was carried out. As indicated in Fig. 6d, incubation with APE and rosiglitazone for 4 h significantly increased PPARγ mediated transcription.

## Discussion

Many fruits and vegetables have traditionally been used in the treatment of diabetes, particularly those with elevated concentrations of polyphenols [4]. In a Finish cohort study [19] examining the association between flavonoid intake and the risk of several chronic diseases, a reduced risk of type 2 diabetes was associated with apple consumption. Moreover, different studies have investigated the effects of polyphenols describing their role in glucose absorption in the gut. Nonetheless, although the potential antidiabetic effect of APE has been previously reported, the molecular mechanisms involved have not been totally elucidated.

The primary purpose of this study was to evaluate whether the hypoglycemic action of apple polyphenols could be mediated, not only by reducing the postprandial glycemic response as consequence of the inhibition of intestinal glucose absorption, but also by enhancing muscle insulin sensitivity. The effects of APE in differentiated L6 rat skeletal muscle cells were also evaluated in order to elucidate the muscle insulin sensitivity mechanisms. These cells have been widely used to investigate the mechanism of insulin-stimulated glucose transport [15, 20] and to gain insight into the related glucose uptake signaling pathways [21].

The results of our study showed that acute administration of APE reduced the glycemic postpandrial response as a consequence of the inhibition of intestinal glucosidases activities. The extract assayed in this study presents a potent α-glucosidase inhibitory action. These results are in agreement with other studies demonstrating that plant polyphenols play an important role in the regulation of the activity of α-glucosidases. Moreover, they agree with a study in which the antihyperglycemic effects of polyphenols from different apple varieties showed that apple polyphenols reduced intestinal glucose absorption through the inhibition of α-glucosidase activities [22].

In addition to the immediate acute effect, APE was evaluated for the second-meal effect on the postprandial glycemic response. The second-meal effect is a parameter commonly evaluated in studies involving natural ingredients or products in order to assess the influence of these compounds over the glycemic response of subsequent meals [23, 24]. In this study, APE administration at the first meal (breakfast) produced a reduction of over 22 % in the glucose response after an oral administration of maltodextrin that simulated a lunch meal, as compared to the control group. The exact mechanism underlying the second-meal effect has yet to be understood and could be related to several mechanisms. Independent of the factors possibly influencing the second-meal effect, insulin sensitivity common improves, especially when the duration of the second-meal effect is not long, e.g. from breakfast to lunch [25].

In order to determine the potential insulin sensitivity effect of APE, the long term effects of APE intake (128 mg/kg bw) were also evaluated after 4 weeks of treatment. Animals that had received APE in fasting conditions showed lower glucose and insulin levels than the control group. Additionally, they had reduced glycemic and insulinemic responses after a challenge with MD (1 g/kg bw). These results are in agreement with another study which used a different model of diet-induced obesity in Wistar rats that were fed apple polyphenols for 8 weeks at the dose of 700 mg/kg bw [26]. In this study, the administration of APE improved glucose tolerance and protected against body weight gain and fat deposition in the rats. The authors of this study suggested that the effects of APE were exerted through the regulation of adipogenesis, lipolysis and fatty acid oxidation. However, based on our results we also suggest that APE may improve glucose tolerance through the stimulation of insulin sensitivity. To prove this hypothesis, insulin sensitivity was evaluated by the euglycemic clamp technique, which is considered the “gold standard” for assessing whole body insulin sensitivity. Our results showed that the 5-week nutritional intervention with APE induced an increase in GIR of 45 %, which is indicative of a better ability of the peripheral tissues to respond to glucose uptake upon insulin stimulation.

At present, it is known that impaired insulin sensitivity and increased insulin resistance are profoundly involved in the progression of type 2 diabetes; and therapies improving or enhancing insulin sensitivity are being sought.

Subsequently, the next step was to investigate the underlying mechanism involved in the stimulation of insulin sensitivity with special focus on muscle as the main tissue responsible for global insulin sensitivity [27].

APE was able to stimulate glucose uptake in L6 myotubes after 18 h of incubation. This effect was dose dependent and additional APE showed a synergistic effect of insulin on glucose uptake. Therefore, treatment with APE could enhance insulin action improving blood glucose levels and preventing insulin resistance.

The increase of glucose uptake observed after incubating cells with APE might have been due to an enhancement of the facilitated translocation of the GLUT4 transporter from the low density microsomes to the plasma membrane by APE. It has been demonstrated that translocation of the glucose transporter GLUT4 to the plasma membrane is a prerequisite for the stimulation of glucose uptake in muscle [20]. This is the first report that has demonstrated that the increase in glucose uptake mediated by APE promotes GLUT4 translocation to the plasma membrane. The results provide evidence that APE may improve hyperglycaemia in type 2 diabetes because skeletal muscle is the major tissue responsible for glucose uptake in response to insulin [28, 29].

Next, the analysis of the signaling pathways involved in the increased GLUT4 translocation mediated by APE was conducted using specific inhibitors. We found that PI3K, a key kinase involved in muscle insulin signaling [30], and PPARγ signaling pathways are responsible for the increase in glucose uptake by APE in L6 myotubes. On the one hand, it has been described that overexpression of PPARγ affects insulin sensitivity of skeletal muscle cells, upregulating glucose uptake under insulin-resistant conditions [31]. On the other hand, similar to APE, other natural extracts, such as *Salacia oblonga*, *Artemisia princeps* and Propolis extracts, have presented an antihyperglycemic effect through activation of PI3K and induction of GLUT4 translocation to the plasma membrane in L6 myotubes [32–35].

The experiment using specific inhibitors of signaling pathways suggested the involvement of the Akt and PPARγ pathways in the effects of APE on glucose homeostasis. Thus, the phosphorylation status of Akt was determined in the presence of APE. Our results confirm the involvement of this pathway. Furthermore, western blot and reporter analysis also confirmed the involvement of PPARγ signaling on APE positive effects. In summary, using an *in vitro* model, we have shown that APE improved glucose uptake in L6 myotubes through a synergistic effect between APE and insulin and an increase in the GLUT4 translocation. Taking into account that GLUT4-dependent glucose transport in skeletal muscle is likely the major mechanism for dietary glucose disposal by skeletal muscle and the increased GLUT4 at the cell surface is associated with an enhancement of insulin sensitivity [36], these results signal that APE could increase muscle-body insulin sensitivity.

## Conclusions

In conclusion, APE exhibited an insulin sensitizing effect activating glucose transport through translocation of GLUT4 mediated by PI3K and, PPARγ signaling pathways in muscle cells. Considered together, APE might emerge as a promising nutritional ingredient to be considered for incorporation in nutritional products focused on the management of chronic diseases such as diabetes.

### Availability of data and materials

Authors do not wish to share their data due to company policy. If any scientist would like more information, please request by email to the corresponding author.

## Abbreviations

2-DG: 2-Deoxy-[3H]D-glucose
APE: apple polyphenol extract
DMEM: Dulbecco’s modified Eagle’s medium
ERK: extracellular signal-regulated protein kinase
GIR: glucose infusion rate
GLUT: glucose transporter
MD: maltodextrin
MTT: meal tolerance test
OZR: obese Zucker rats
PI3K: phosphatidil inositol 3-kinase
PPARγ: peroxisome proliferator-activated receptor-gamma
SEM: standard error of the mean
SGLT: sodium-glucose linked transporter
